# Fibroblast growth factor 2 supports osteoblastic niche cells during hematopoietic homeostasis recovery after bone marrow suppression

**DOI:** 10.1186/s12964-017-0181-2

**Published:** 2017-06-29

**Authors:** Kyung-Ae Yoon, YeonSung Son, Young-Jin Choi, Joo-Hyun Kim, Je-Yoel Cho

**Affiliations:** Department of Biochemistry, BK21 PLUS Program for Creative Veterinary Science Research and Research Institute for Veterinary Science, College of Veterinary Medicine, Seoul National University, 1 Gwanak-ro, Gwanak-gu, Seoul, South Korea

**Keywords:** FGF2, Hematopoietic stem cell, Niche, Osteoblast, Osteoprogenitor

## Abstract

**Background:**

Hematopoietic stem cell (HSC) maintenance requires a specific microenvironment. HSC niches can be activated by tissue damaging chemotherapeutic drugs and various cell signaling molecules such as SDF-1 and FGF, which might also result in bone marrow stress. Recent research has insufficiently shown that endosteal osteolineage cells and other niche constituents recover after marrow injury.

**Methods:**

We investigated the role of FGF2 in the osteoblastic niche cells during hematopoietic homeostasis recovery after bone marrow injury. Mice were treated with 5-fluorouracil (5FU) to eliminate actively cycling cells in the bone marrow. Primary osteoblasts were isolated and subjected to cell culture. Real-time PCR, western blot and immunohistochemical staining were performed to study niche-related genes, osteoblast markers, and FGF2 signaling. Proliferation rate were analyzed by marker gene Ki67 and colony formation assay. Also, osterix-positive osteoprogenitor cells were isolated by FACS from Osx-GFP-Cre mice after 5FU treatment, and subjected to RNA-sequencing and analyzed for Fgf receptors and niche markers.

**Results:**

The endosteal osteolineage cells isolated from 5FU-treated mice showed increased expression of the niche-related genes *Sdf-1*, *Jagged-1*, *Scf*, *N-cad*, *Angpt1* and *Vcam-1* and the osteoblast marker genes *Osx*, *Opn*, *Runx2*, and *Alp*, indicating that BM stress upon 5FU treatment activated the osteoblastic niche. Endosteal osteoblast expanded from a single layer to several layers 3 and 6 days after 5FU treatment. During the early recovery phase in 5FU-activated osteoblastic niches increased FGF2 expression and activated its downstream pERK. FGF2 treatment resulted in increased proliferation rate and the expression of niche marker genes in 5FU-activated osteoblastic niche cells. RNA-seq analysis in Osterix-positive osteoprogenitor cells isolated from 5FU-treated Osx-GFP mice showed significantly increased expression of *Fgf* receptors *Fgfr1, 2 and 3*. Although osteoblastic niche cells were damaged by 5FU treatment in the beginning, the increased number of OB layers in the recovery phase may be derived from resident osteoprogenitor cells by FGF2 activation under stress.

**Conclusions:**

Taken together, FGF2 signaling can regulate osteoblastic niche cells to support HSC homeostasis in response to bone marrow damage.

## Background

Hematopoietic stem cells (HSCs) are cells capable of differentiating into all blood cell lineages, and they are tightly regulated by the local niche to maintain homeostasis. HSC niches can be compromised by several different cell types, including endosteal osteolineage cells and vascular sinusoidal endothelial cells in the bone marrow. Regulatory signal emanating from surrounding cells within the niche and physiologic cues could affect HSC quiescence, proliferation and differentiation [1, 2]. Under steady state conditions, the majority of HSCs are quiescent but can be activated to proliferate and differentiate after suppression by infection, radiation, or chemotherapeutic drug treatment, such as cyclophosphamide and 5-fluorouracil (5FU) [3–6]. Chemotherapeutic drug treatment for hematological diseases induce damage to the bone marrow (BM) microenvironment through multiple BM stress responses. Under BM stress, cycling cells are eliminated and surviving quiescent long-term hematopoietic stem cells (LT-HSCs) are activated to expand via a complex mechanism [7–9].

Fibroblast growth factors (FGFs) are secreted molecules that regulate a variety of biological processes, such as cell proliferation, differentiation, and migration during both embryonic and adult development [10]. FGFs bind FGF receptors (FGFRs) and heparin sulfate proteoglycans (HSPGs) to modulate downstream biological processes. FGFRs include 4 tyrosine kinases designated FGFR1–4. Interactions of FGF and the receptor display different ligand-binding and biological functions [11]. The various subtypes of FGFRs form via splicing in immunoglobulin domain three of FGFR1, FGFR2 and FGFR3, but not FGFR4. Therefore, there are several FGFR1-3 subtypes, such as FGFR1-3 IIIb and FGFR1-3 IIIc.

Several studies have suggested that FGF signaling has important roles in HSC maintenance. FGF2 knock-out (KO) BM stromal cells weakly support HSC maintenance [12]. Under irradiation conditions, FGF2 KO mice showed delayed recovery of BM LSK (Lin^−^Sca-1^+^cKit^+^) HSPC cells and a reduced population of CD34^−^LSK cells (enriched with LT-HSCs) [13]. The microenvironment indirectly mediates FGF signaling, as HSCs expanded only in unfractionated BM culture [14]. BM suppression induced HSC activation by FGF secreted by megakaryocytes [9]. While it is known that osteoprogenitor cells have important roles in HSC maintenance, the endosteal osteoblasts (OBs) significantly expanded upon BM stress [15–17]. FGF signaling in bone is related to skeletal development and disease [18]. Deletion of the niche factor SDF-1 using osterix (Osx)-cre led to significant HSC mobilization [19]. Deletion of Osx in bone also affects hematopoiesis in the BM [20].

Here, we studied whether FGF2 could support osteoprogenitor cell-derived endosteal osteoblastic cells to recover HSPCs and modify the microenvironment to support HSPC recovery after 5FU-induced stress. The results showed that FGF2 mediates endosteal osteoblastic niche cell proliferation upon 5FU-induced stress. Activated FGF signaling increased pERK levels early after acute injury and increased *Fgfr* expression in the OB niche cells. In particular, our study suggests that, after BM stress, Osx-positive osteoprogenitor cells are activated by increasing *Fgfr* expression to proliferate in response to FGF2.

## Methods

### Animals and 5FU treatment

C57BL/6 mice were used to isolate primary osteoblasts, and the hematopoietic stem cell niche was activated by 5-fluorouracil (5FU) (Sigma-Aldrich, St. Louis, MO, USA). Mice were injected once with 5FU at a dose of 150 μg/g body weight. Bone tissues were harvested to isolate primary osteoblasts (endosteal osteolineage cells) at various time points after 5FU treatment. Osx-GFP-Cre mouse were purchased from The Jackson Laboratory.

### Primary osteoblast isolation

Primary osteoblast cells were isolated as previously reported by our group [21]. Briefly, crushed bones (femora, tibiae, and humeri) were removed from control or 5FU-treated mice and washed with PBS (GIBCO, Grand Island, NY) with 2% FBS (Hyclone, South Logan, UT) until the bone chips were white. Endosteal osteoblasts were released by enzyme digestion for 90 mins at 37 °C at 150 rpm with 3 mg/ml type I collagenase (Worthington, Lakewood, NJ) and 0.05% dispase (GIBCO) in PBS with 20% FBS. The released endosteal osteoblasts were washed in PBS + 2% FBS, and after removing the residual bone material osteoblasts were made into a single cell suspension by filtering through a 45-μm filter (BD Bioscience, San Jose, CA). In some experiments, osteoblastic cells were also isolated by the explant culture method in a 12-well plate containing the same weight of bone tissues per well. After primary osteoblasts became confluent within 5 to 10 days in the presence of FGF2 (R&D systems, Minneapolis, MN) or without FGF2, osteoblast colonies originating from bone fragments were washed with PBS (phosphate-buffered saline) and stained with a methanol-crystal violet solution (0.4%; wt/vol). Bones from non-5FU-treated mice were processed as a control for osteoblast growth recovery.

### Cell culture

Primary osteoblasts were seeded in a 12-well plate. To investigate the effect of FGF2 in primary osteoblasts from 5FU-treated mice, FGF2 (50 or 80 ng/ml) was added to cultured primary osteoblasts for 15 days. The FGFR inhibitor SU5402 (Sigma-Aldrich) was added to the culture medium at a final concentration of 5 μΜ.

### RNA isolation and real-time RT-PCR

Total RNA was isolated from primary osteoblasts using TRIzol reagent (Invitrogen, Carlsbad, CA) according to the manufacturer’s protocol. Total RNA (1–1.5 μg) was reverse-transcribed using the Omniscript Reverse Transcription kit (QIAGEN, Valencia, CA). mRNA expression levels were measured either by conventional PCR or by fluorescence-based real-time PCR. Quantitative real-time PCR using CFX Connect™ Real-Time PCR system (Bio-Rad, Hercules, CA) or conventional PCR using Go-taq® Flexi DNA polymerase (Promega, Madison, WI) was performed in a volume of 15 μl 1× SYBR^®^ green premix Ex Taq™ (TAKARA BIO Inc., Shinga, Japan) and 20 pM forward and reverse primers (Bioneer, Daejeon, Republic of Korea). To normalize for input RNA, murine *Gapdh* (Glyceraldehyde-3-phosphate dehydrogenase) was amplified as an endogenous control. Amplification conditions were as follows: 95 °C for 5 mins followed by 40 cycles of 95 °C for 20 s, and annealing temperature of each primer for 30 s. The melting curve protocol was performed for each primer set to confirm specificity. All samples were run in triplicate. The primer sequences for each gene are shown in Table 1.

**Table 1:** Gene primers list

Target gene	Primer sequence	
	Forward (5′-3′)	Reverse (5′-3′)
*Gapdh*	GGCATTGCTCTCAATGACAA	TGTGAGGGAGATGCTCAGTG
*Alp*	GATAACGAGATGCCACCAGAGG	TCCACGTCGGTTCTGTTCTTC
*Runx2*	AGATGATGACACTGCCACCTCTG	GGGATGAAATGCTTGGGAACTGC
*Opn*	TGCACCCAGATCCTATAGCC	CTCCATCGTCATCATCATCG
*Osx*	CTCTCTGCTTGAGGAAGAAG	GTCCATTGGTGCTTGAGAAG
*Ki67*	CAGACGAGCAAGAGACAAAG	AAGTACGGAGCCTGTATCAC
SDF-1	GCTCTGCATCAGTGACGGTA	TAATTTCGGGTCAATGCACA
*Jagged-1*	CTTCAATCTCAAGGCCAGCC	CAGGCGAAACTGAAAGGCAG
*N-cadherin*	ACGAGAGGCCTATCCATGCTGAGC	TCAGTCGTCACCACCGCCGTACAT
*Vcam-1*	ATTTTCTGGGGCAGGAAGTT	ACGTCAGAACAACCGAATCC
*Angpt1*	AGGCTTGGTTTCTCGTCAGA	TCTGCACAGTCTCGAAATGG
*Fgfr1IIIb*	CTTGACGTCGTGGAACGATCT	AGA ACGGTCAACCATGCAGAG
*Fgfr1IIIc*	AGAGACCAGCTGTGATGACC	TGTTACCTGTCTGCGCAGAG
*Fgfr2IIIb*	CCCATCCTCCAAGCTGGACTG	CAGAGCCAGCACTTCTGCATTG
*Fgfr2IIIc*	CCCATCCTCCAAGCTGGACTG	TCTCACAGGCGCTGGCAGAAC
*Fgfr3IIIb*	GGCGCTAACACCACCGAC	TGGCAGCACCACCAGCCAC
*Fgfr3IIIc*	TGGAAACTGATGAGGCTGGC	AAACGAGAGACCTTGTGCAC

### Western blotting

Western blot analysis was performed as previously reported [21]. Briefly, primary osteoblasts isolated from PBS- or 5FU treated mice were lysed in lysis buffer (RIPA buffer, 50 mM Tris-Cl [pH 8.0], 150 mM NaCl, 1% NP-40, 5 mM EDTA, 1 mM PMSF; Thermo Scientific, Rockford, IL) containing a protease cocktail (Roche, Mannheim, Germany) and phosphatase cocktail. Equal amounts of cell lysate protein were subjected to SDS-PAGE and transferred to nitrocellulose membranes (Whatman, GE healthcare Bioscience, Pittsburgh, PA). After blocking, the membranes were incubated with primary antibodies (phospho-p44/p42 MAPK (Erk1/2) (1:3000) (Cell Signaling, Beverly, MA) and ERK (1:500) (Santa Cruz Biotechnology, Inc., Dallas, TX), overnight at 4 °C and then incubated with the appropriate horseradish peroxidase-conjugated secondary antibody for 1 h at RT. The blots were developed using a chemiluminescence detection system (ECL kit; Amersham Pharmacia Biotech, Piscataway, NJ) and exposed to x-ray film (AGFA, Mortsel, Belgium).

### Harris hematoxylin and eosin staining

The femora and tibiae of PBS- or 5FU- treated mice were fixed in 4% paraformaldehyde phosphate buffer solution (Wako, Osaka, Japan). Bones were decalcified in 0.5 M EDTA (ethylenediaminetetraacetic acid) (Welgene, Daejeon, Republic of Korea) for 3 weeks before dehydration, paraffin-embedded, and cut into 5- to 7-μm sections. For osteoblast analysis in 5FU treated mice, sections were stained with Harris hematoxylin and eosin (Sigma-Aldrich).

### RNA-sequencing analysis

Total RNA was isolated for analyze RNA-sequencing using RNeasy^®^ PLUS Micro (QIAGEN) according to the manufacturer’s protocol. The RNA-sequencing library was prepared using an Illumina TruSeq RNA sample prep kit and quantified using a KAPA library quantification kit. Total library fragments were used to generate clusters, followed by sequencing on an Illumina HiSeq 2500. Gene expression was quantitated using Cufflinks v2.1.1.

### Immunohistochemical analysis

For immunohistochemistry analysis, the bone tissue (femur and tibiae) were harvested from PBS- and 5-FU-treated mice on 3 days and 6 days and processed for immunohistochemistry [22]. Tibiae were fixed in 4% paraformaldehyde (PFA) and paraffin-embedded and cut into 6-μm sections. After dewaxing and rehydrating, endogenous peroxidase was quenched for 15 min with 3% H_2_O_2_ in methanol. Heat-mediated antigen retrieval and enzymatic techniques were performed according to recommendations for the specific antibodies. A blocking step was performed using 10% normal goat serum and 1% bovine serum albumin (BSA) in PBS. After endogenous peroxidase and nonspecific protein block, primary antibodies were incubated overnight at 4 °C as follows: anti–FGF2 (Santa Cruz, CA, USA) primary antibodies. Polyclonal secondary antibodies were incubated, followed by incubation in streptavidin horseradish peroxidase (Invitrogen, CA, USA). Staining was developed with DAB according to manufacturer’s instructions (Invitrogen, CA, USA) and briefly counterstained in methy green before coverslipping in cytoseal permanent mounting media. Localization of positive staining was analyzed by light microscopy (Olympus CX41, Japan).

### Statistical analyses

Data are presented as means ± SD, and statistical comparisons between groups were performed by one-way analysis of ANOVA test. * and ** symbols indicate *p* < 0.05 and *p* < 0.01 vs control group, respectively.

## Results

### Bone marrow suppression increases HSC niche related-genes in osteoblasts

**Fig. 1:**
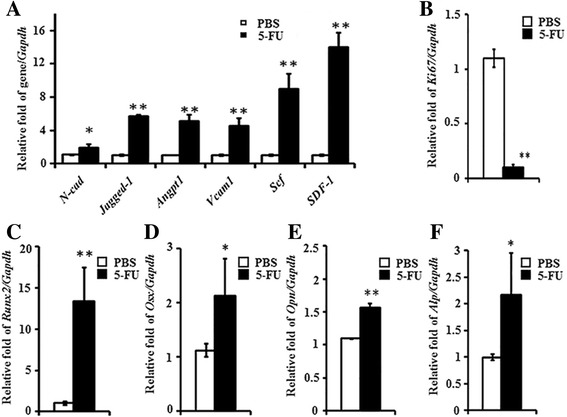
HSC niche related-genes and bone marker genes in primary endosteal osteoblasts isolated from 5FU-treated mice. Total RNA was isolated from the osteoblasts of the mice treated with PBS (*n* = 5) or 5FU (*n* = 5) for 4 days. **a**, **b** The mRNA expression levels of SDF-1, *N-cad*, *Jagged-1*, *Angpt1*, *Vcam1*, *Scf* (**a**) and *Ki67* (**b**) related to *Gapdh* levels were analyzed by qRT-PCR and presented as ratios to the control (PBS) OBs’ relative value. **c**-**f** The mRNA expression levels of *Runx2* (**a**), *Osx* (**b**), *Opn* (**c**) and *Alp* (**d**) related to *Gapdh* levels were analyzed by qRT-PCR and presented as ratios to the control (PBS)

The 5FU treatment eliminates actively cycling cells in the BM and induces surviving quiescent LT-HSC activation. We first investigated whether the osteoblastic niche in 5FU-treated mice could change to support HSC reconstitution in response to this BM damage. After administering a sublethal dose of 5FU to the mice, we observed an increase in HSC niche-related gene expression in primary osteoblasts isolated from mouse long bones (femurs, tibiae and humeri). HSC niche-related genes, *N-cad*, *Jagged-1*, *Angpt1*, *Vcam-1*, *Scf*, and *sdf-1* significantly increased in the osteoblasts isolated from 5FU-treated mice for 4 days compared to control osteoblasts (Fig. 1a). However, the proliferation marker gene *Ki67* was highly decreased in the OB after 4 days of 5FU treatment (Fig. 1b). It was previously shown that osteocalcin- and collagen I-positive cells increased in trabecular bones of the metaphysis of irradiated mice, and the mRNA expression of niche marker genes increased in endosteal osteoblasts isolated from irradiated mice [16]. These results suggest that the osteoblast lineage cells at the endosteal surface change to be activated as niche cells after 5FU-induced BM damage.

### Bone marrow suppression also increases osteoblast marker genes expression

Since it is known that early stage osteoblast cells serve as HSC niche cells [23], we also examined whether osteoblast marker genes are increased upon 5FU treatment. It was previously shown that endosteal osteoblasts of irradiated mice increased osteocalcin and collagen type I expression and that hematopoietic cell clusters closely contacted to the bone (endosteal osteoblast zone) after 5FU treatment [16, 24]. In our study, the early osteoblast marker genes *Runx2* and *Osx* were increased in osteoblasts of 5FU-treated mice compared to control (Fig. 1c and d). *Alp* and *Opn* expressions were also increased (Fig. 1e and f). These data suggest that 5FU-induced BM damage leads to the activation of niche cells at early stage of osteoblast in the BM.

### 5FU-damaged bone marrow induces decreased proliferation rates of endosteal osteoblasts

**Fig. 2:**
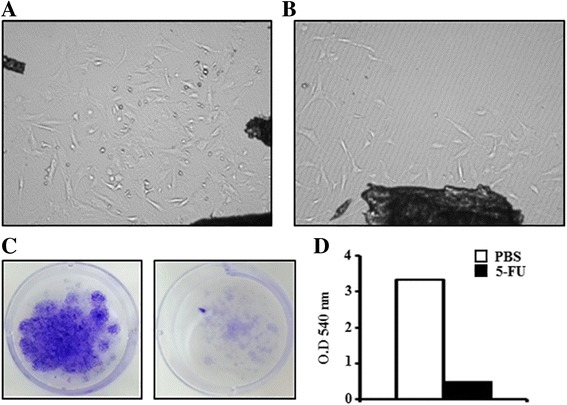
Proliferation rate of endosteal osteoblasts isolated from 5FU-treated mice. Representative photographs of in vitro-cultured OBs selectively obtained from bones in PBS- (**a**) or 5FU-treated mice (**b**) for 4 days (*n* = 5). Long bone fragments completely depleted of bone marrow were cultured in α-MEM medium containing 15% FBS for 14 days. **c** & **d** Representative pictures of OB colonies from PBS- or 5FU-treated mice after 14 days of culture CFU assay (**c**), and O.D. values at 540 nm (per 44 mg of processed bone chip) after 2 weeks of culture of OBs from PBS- or 5FU-treated mice (**d**)

To determine whether 5FU treatment affected endosteal osteoblast survival and proliferation, we maintained OBs selectively isolated from long bone explants of 5FU-treated mice in vitro. Figure 2a and b show photographs of in vitro-cultured OBs for 2 weeks from the bone of PBS- or 5FU-treated mice for 4 days. OBs obtained from 5FU-treated mice showed a reduction in proliferation rate compared to the control. We also counted OB colonies at 2 weeks by CFU-F assay and measured the O.D. value of OB colonies from 5FU-treated mice and control mice (Fig. 2c and d). 5FU treatment-induced bone marrow suppression not only eliminated actively cycling cells in the BM but also affected osteoblast proliferation.

### Fibroblast growth factor 2 supports the proliferation rate and increases HSC niche-related genes expression in endosteal osteoblasts of 5FU-treated mice.

**Fig. 3:**
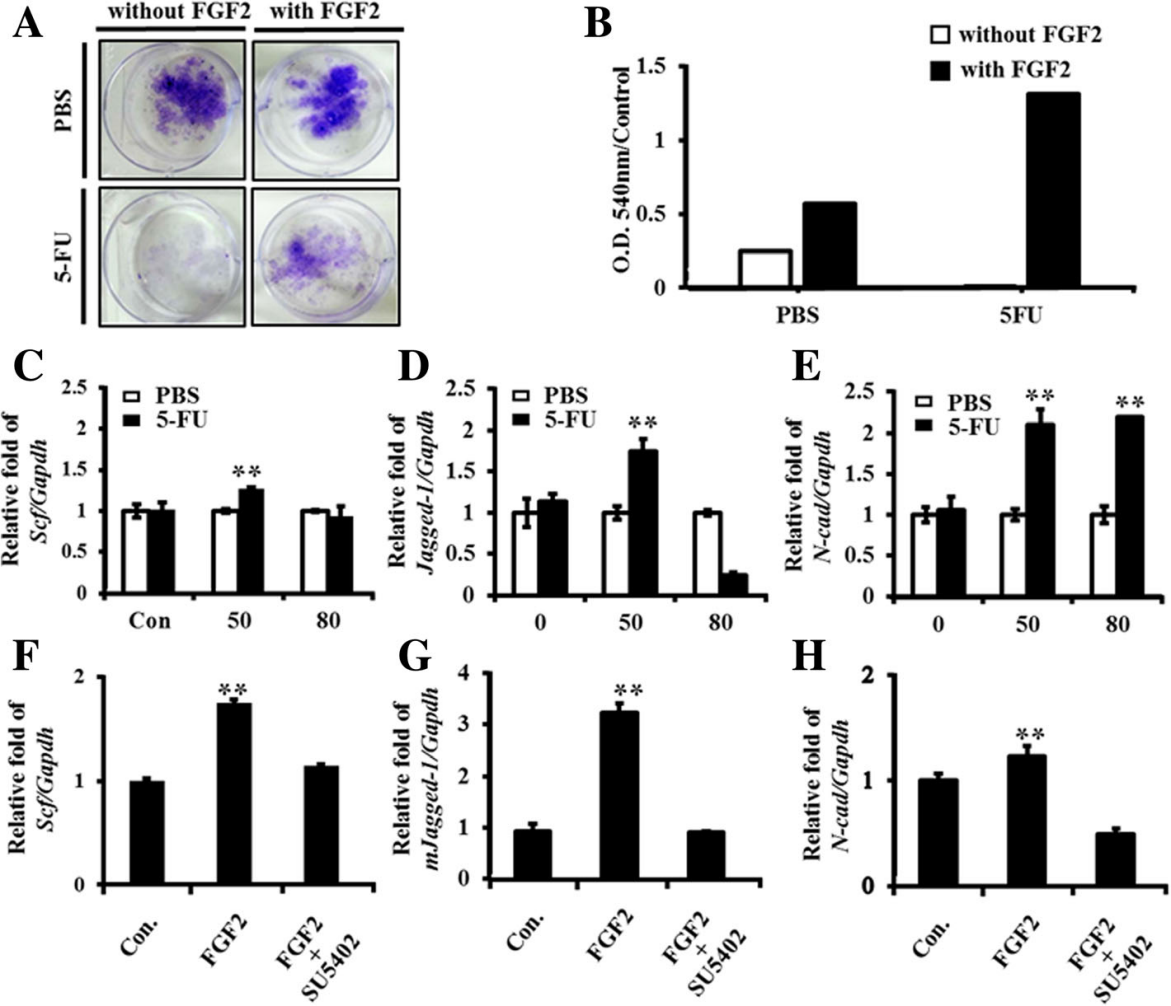
The proliferation rate and expression of HSC niche-related genes increased by FGF2 in endosteal osteoblasts isolated from 5FU-treated mice. **a** & **b** Representative pictures of OB colonies from PBS- or 5FU-treated mice for 4 days, after culture in the presence or absence of FGF2 (50 ng/ml) for 14 days. CFU assay was performed and O.D. values were measured at 540 nm. The O.D value was normalized to the bone weight used for primary cell culture and was presented as a relative O.D. values to the control of ‘without FGF2’ (thus expressed ‘O.D. 540 nm/control’) (**c**, **d**, **e**) The PBS- or 5FU-activated OBs were cultured in the presence of FGF2 at various concentrations (0, 50, 80 ng/ml) for 14 days. Total RNA was isolated, and the expression of HSC niche-related genes *Scf, Jagged-1, N-cad* was analyzed compared to *Gapdh* levels by qRT-PCR and presented as ratios to the control (PBS) OBs’ relative value. **f**, **g**, **h** Blocking of FGF receptor signaling by FGFR inhibitor. 5FU-activated OBs were cultured from 5FU-treated mice for 4 days in the absence or presence of SU5402 (5 μM) for 14 days. Total RNA was isolated and the expression of HSC niche-related genes *Scf* (**f**), *Jagged-1* (**g**), and *N-cad* (**h**) were analyzed against *Gapdh* levels by qRT-PCR and presented as ratios to the control OBs’ relative value

We have shown that acute injury by 5FU to suppress bone marrow cycling cells also affected the endosteal osteoblastic niche cell proliferation ex vivo (Fig. 2). Therefore, we hypothesized that endosteal osteoblastic niche cells should be activated by various factors, including cytokines and growth factors for the niche cells to be activated to support HSPCs and to allow for damaged BM to recover after acute injury. Recently, mature megakaryocytes (MMs) residing within BM have been shown to directly and indirectly influence various cells in the BM, including HSCs and OBs. MMs stimulate their proliferation and differentiation through the regulation of OB function [25–27]. MMs are known to be a major source of factors, including insulin-like growth factor (IGF) -1 and fibroblast growth factor 2 (FGF2), that are important in OB regulation and recovery in damaged BM [9, 15, 16]. We investigated the effect of FGF2 on osteoblastic niche cell proliferation and function in endosteal OBs from 5FU-treated mice. The primary cells from the mice were cultured for 2 weeks with or without FGF2 (50 ng/ml) and OB colonies were examined by crystal violet staining and measured the O.D. value of the colonies in cultured OBs from 5FU-treated mice and control. The colony formation in CFU-F assay and the O.D. values (normalized to the bone weight used for primary cell culture and compared to ‘without FGF2’ control (O.D. 540 nm/control)) were significantly increased (126-fold) in FGF2-treated OBs from 5FU-treated mice while the colonies were mildly increased in PBS control OBs by FGF2 treatment (Fig. 3a and b).

The expressions of HSC niche-related genes, *Scf, Jagged-1* and *N-cadherin,* were increased in FGF2 (50 ng/ml) -treated OBs (Fig. 3c-e). However, although *N-cad* mRNA expression was significantly increased in 50 ng/ml FGF2 treatment (Fig. 3e), *Jagged-1* level was decreased by high concentration of FGF2 (80 ng/ml) (Fig. 3d). In addition, FGFR inhibitor SU5402 (5 μM) treatment resulted in the reduction of these niche-related *Scf*, *Jagged-1*, and *N-cadherin* gene expression from the increase of FGF2 to almost basal levels (Fig. 3f-h). These data highlight the importance of FGF2 signaling in the regulation of HSC niche-related genes upon BM suppression.

### *Fgfrs* subtype expression in endosteal osteoblasts

**Fig. 4:**
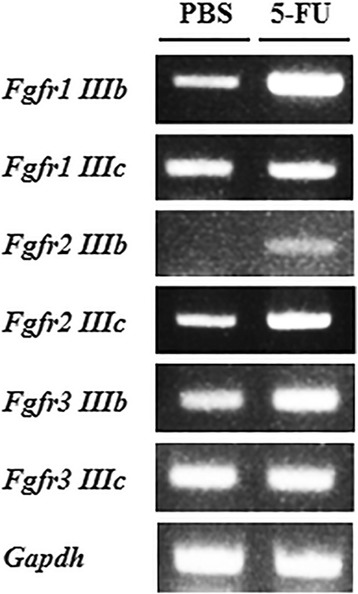
Expression of *Fgfr* genes in the endosteal osteoblasts isolated from 5FU-treated mice. Total RNA from PBS- or 5FU-activated OBs for 4 days was subjected to RT-PCR. Expression of *Fgfr* genes in these cells was confirmed using primer pairs by PCR. PCR products were separated on a 1.5% agarose gel containing ethidium bromide (EtBr) and visualized under UV light. Representative results from at least 3 independent experiments are shown

We have shown that FGF2 supports osteoblastic niche cells, and increases HSC niche-related gene expression and the proliferation rate of OBs from 5FU-treated mice to allow for the recovery of BM suppression. To confirm FGF signaling activation in the endosteal OBs under stress, we performed PCR analysis to examine *Fgfr* expression in the endosteal OBs. The endosteal OBs expressed *Fgfr1*–*3,* including all subtypes except *Fgfr2IIIb* and *Fgfr4* (Fig. 4). Surprisingly, the expressions of *Fgfr1IIIb, Fgfr2IIIb, Fgfr2IIIc,* and *Fgfr3IIIb* were increased after 5FU stress. These results suggest that *Fgfrs* were activated to receive more FGFs signal in the OBs of 5FU-treated mice.

### Expression of *Fgfrs* and the proliferation marker gene *Ki67* in the osteoprogenitor (osterix-positive) cells of 5FU-treated mice

**Fig. 5:**
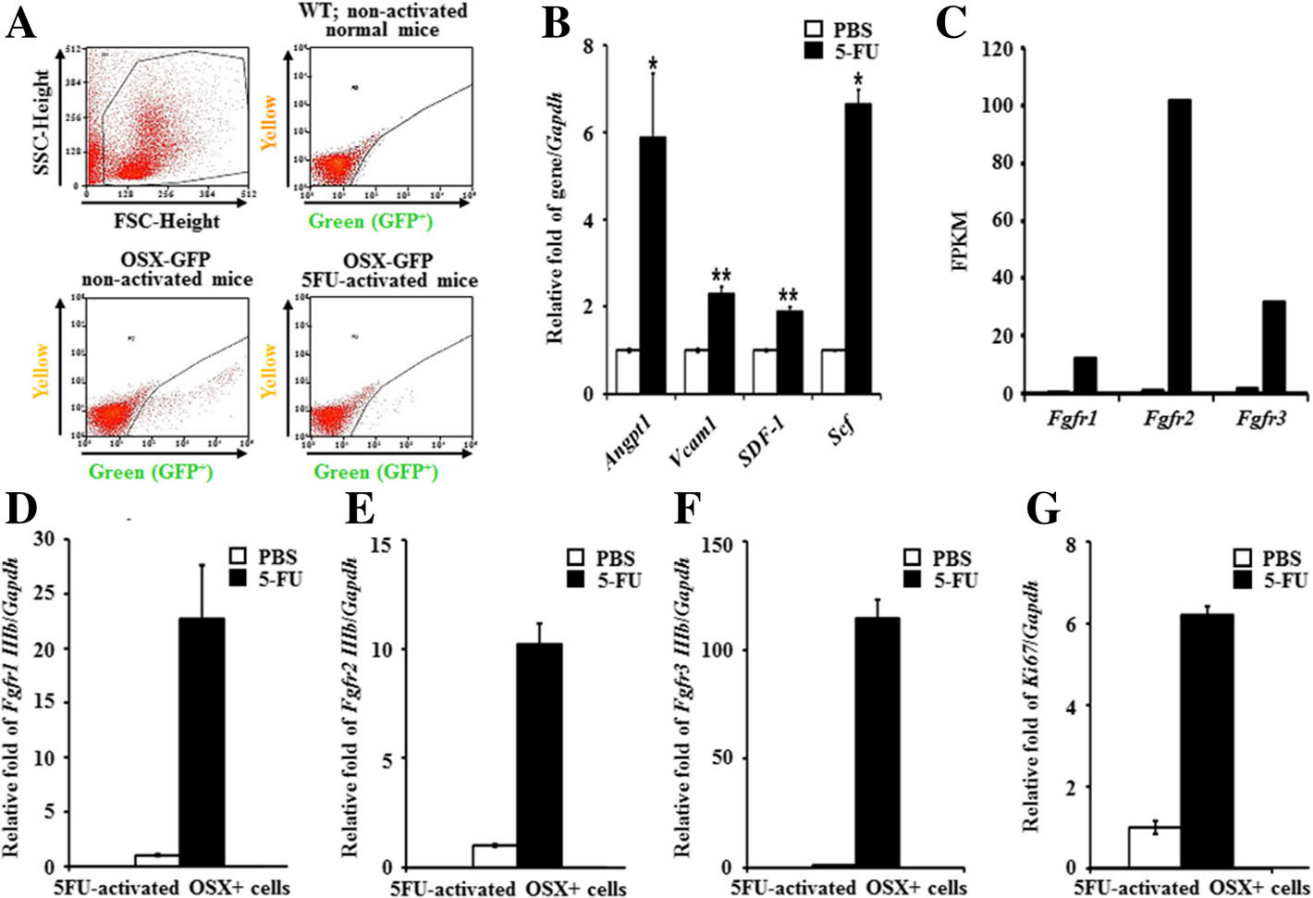
FGF signal and HSC niche-related genes were activated in osteoprogenitor cells (Osx+ cells) isolated from 5FU-treated Osx-GFP mice. **a** FACS analysis to sort GFP-positive cells from Osx-GFP mouse treated with 5FU for 4 days. **b** Total RNA was isolated from PBS- or 5FU-activated Osx positive cells (osteoprogenitor cells). mRNA expression levels of SDF-1, *Angpt1*, *Vcam1* and *Scf* related to *Gapdh* levels were analyzed by qRT-PCR and presented as ratios to the control (PBS) Osx positive cells’ relative value. **c** RNA-seq analysis of PBS- or 5FU-activated Osx+ cells for *Fgfrs*. Expression level shown by FPKM (Fragments per kilobase of exon per million fragments mapped). **d**, **e**, **f** Gene expression of Fgfr1–3 using qRT-PCR of Osx+ osteoprogenitor cells from PBS- or 5FU-treated mice for 4 days. **﻿g** Expression of *Ki67 *gene in Osx+ progenitor cells from PBS- and 5FU-treated mice for 4 days

The BM environment is composed of multiple cell types, including endosteal osteoblastic niche cells and osteolineage cells, which support HSPC homeostasis. Osteoblast progenitor and mesenchymal stem cells highly express the cytokines SDF-1 and Scf, which are critical for HSC maintenance in mice [19, 28]. *Osx*-positive osteoprogenitor cells are important osteoblastic niche cells, although *Osx*-expressing OBs are restricted short-lived cells in the adult BM [29]. We sorted osteoprogenitor (GFP-positive) cells by FACS from PBS- or 5FU-treated *Osx*-GFP-Cre mice for 4 days to perform RNA-seq (Fig. 5a). Approximately 6000 to 10,000 GFP positive cells were collected from about 20 mice in each group of either control or 5FU activated BMs of Osx-GFP-Cre mice. These GFP+ cells in 5FU treated mice expressed the HSC niche-related genes *Angpt1*, *Vcam1*, *Sdf-1*, and *Scf* higher than the control group (Fig. 5b). RNA-seq analysis on sorted Osx+ cells showed FPKMs (fragments per kilobase of exon per million fragments mapped) for *Fgfr1*, *Fgfr2*, and *Fgfr3* were very high in 5FU-*Osx-*positive cells compared to PBS control (Fig. 5c). Among the 3 *Fgfrs*, *Fgfr2* was more highly expressed than *Fgfr1* and *Fgfr3*. The qRT-PCR of *Fgfr1–3* expressions in Osx-positive cells from 5FU-treated mice after BM suppression showed that *Fgfr1* (10.23-fold), *Fgfr2* (448.8-fold), and *Fgfr3* (114.52-fold) were significantly increased in Osx-positive cells (Fig. 5d-f). *Ki67* expression also increased (Fig. 5g). Increased *Fgfr1–3* and *Ki67* expression indicates that early stage Osx-positive osteoblastic niche cells become highly sensitive to FGF signaling by increasing their FGF receptors and proliferate after BM stress.

### Endosteal osteoblasts activated the ERK pathway and osteoblast expansion after BM suppression by 5FU treatment

**Fig. 6:**
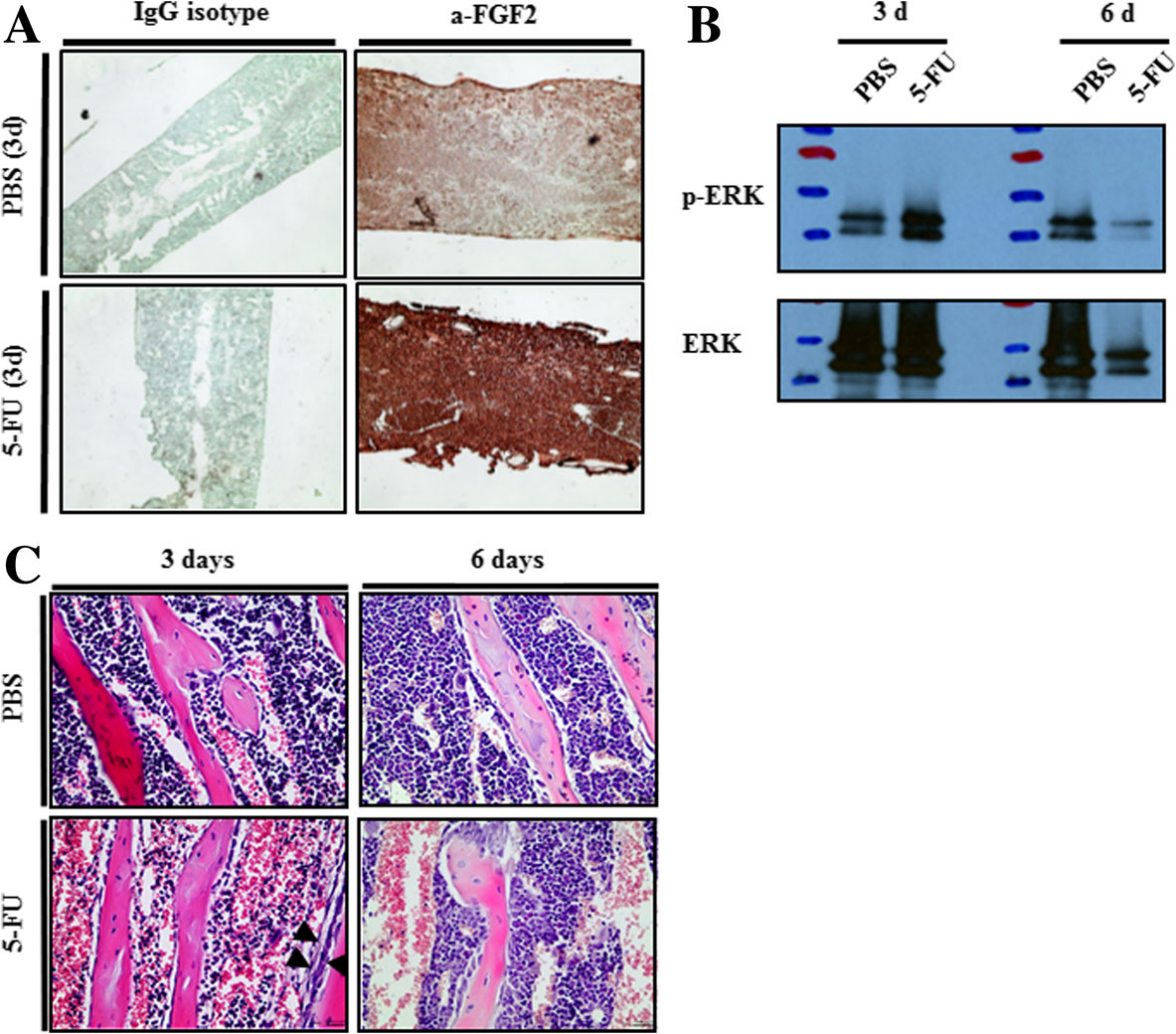
FGF2 increase, ERK pathway activation, and the expansion of endosteal osteoblast population following 5FU treatment. **a** Immunohistochemical staining on the bone marrow of mice 3 days after 5FU treatment. Bone section counterstained with methyl green. Magnification ×100. **b** Total proteins were extracted at 3 and 6 days after 5FU treatment in endosteal OBs from PBS- or 5FU-treated mice. pERK and ERK expression was analyzed by western blotting. **c** Representative H&E stained sections of tibiae at 3 and 6 days following 5FU treatment in mice. Endosteal OBs showed increased cellularity along the endosteal surface at the indicated time points. Arrows indicates multilayer cuboidal osteoblast on the trabecular bone

To test whether the FGF2 levels are affected during the early recovery phase after 5FU treatment, bone marrow tissues were immunostained with anti-FGF2 antibody. Three days after 5FU treatment, protein levels of FGF2 in bone marrow was significantly increased in 5FU treatment compared to PBS control. The staining shows a saturated level of FGF2 in 5FU treated 3-day sample (Fig. 6a). To confirm also whether FGF2 downstream signaling is activated in endosteal OBs following 5FU treatment, phospho-ERK, a downstream of Fgfr activation, was measured by western blot. The phospho-ERK protein levels in endosteal OBs isolated from 5FU-treated mice at 3 days highly increased, (Fig. 6b). These results suggest that BM suppression by 5FU treatment induced these acute injury and FGF2 increase followed by Fgfr-pERK activation in osteoblastic niche cells lasts 3–4 days. Thereafter, pERK signals decrease. Short-term increases of FGF proteins in the BM after 5FU treatment could support osteoblastic niche cells to recover HSPC through FGF signaling.

It was previously shown that osteoblast expansion increased 48 h after radioablative conditions [17]. We investigated whether osteoblast expansion increased to support HSPCs during short-term recovery in the BM after 5FU treatment in mice. Histological analysis showed endosteal osteoblast expansion from a single layer in control (PBS-treated) mice to several layers at 3 days in 5FU-treated mice (Fig. 6c). Expanded endosteal OBs also showed changed morphology from flattened to cuboidal. This morphological changes may increase adhesive interactions between the osteoblastic niche and HSPCs, and migration [30].

## Discussion

The BM and HSC niche support HSC self-renewal and differentiation through interactions of various BM cells and secreted molecule such as FGFs and EGF [9, 31, 32]. The majority of HSCs are quiescent during homeostasis, but these cells can be activated to proliferate and differentiate in response to infectious stress [33]. High dose chemotherapy or radiation treatment for hematological disease such as leukemia or lymphoma may induce damage to the BM HSC niche. Chemotherapy also affects HSC niche cells, such as osteoprogenitor cells, endothelial cells, and megakaryocytes, and induces changes to make an appropriate microenvironment for HSC regeneration [5].

Our data indicate that FGF signaling is important in osteoblastic niche cells, including Osx-positive osteoprogenitor cells, in the recovery following 5FU-induced acute bone injury. These conclusions are based on evidences that (1) HSC niche-related gene expression increased in osteoblastic niche cells isolated from 5FU-treated mice, (2) FGF2 increased proliferation in damaged osteoblastic niche cells, and (3) FGF receptor signaling increased in osteoblastic niche cells, especially Osx + osteoprogenitor cells, following 5FU treatment.

A fundamental question in our studies was whether increased FGFs in the BM following 5FU could recover osteoblastic niche cells to support regeneration of BM suppression. The proliferation marker *Ki67* decreased after 5FU treatment at 4 days and also osteoblastic niche cell proliferation was inhibited in vitro during cell culture (Figs. 1 and 2). However, OB cell proliferation was increased dramatically and niche markers were also increased upon FGF2 treatment in the BM cells from 5FU stressed mice (Fig. 3). It is also of note that recovery phases after 4 days increased the levels of FGF protein in damaged BM and the proliferation of osteoblastic cells in damaged bone through radiation treatment [9, 16]. We also confirmed that the osteoblast marker genes *Runx2*, *Osx*, *Opn*, and *Alp* increased in osteoblastic niche cells at 4 days following 5FU (Fig. 1). In particular, the early osteoblastic markers *Runx2* and *Osx* increased in osteoblasts from 5FU-treated mice. These results corresponded with amplified cellularity along the endosteal surface possibly from endogenous osteoprogenitor cells 3 days after BM suppression (Fig. 6c).

To address the mechanisms of OB survival and proliferation in BM after 5FU treatment in mice, we focused on FGF2, a factor that induces an increase in the OB population and promotes early OB differentiation, because it is known that FGF2 is highly secreted in the BM of 5FU-treated BM-suppressed mice [34, 35]. OBs from 5FU-treated mice significantly reduced the proliferation rate (Fig. 2). These results also indicate that OBs were initially damaged in the BM following 5FU, as shown by decreased *Ki67* expression. Conversely, FGF2 treatment induced the proliferation of OBs damaged by 5FU during in vitro culture (Fig. 3a and b). It also induced increased HSC niche-related gene expression, including *Scf*, *Jagged-1*, and *N-cad* (Fig. 3c-e). N-cad was highly expressed in the endosteal ALCAM^+^SCA-1^−^ cell population, which can enhance LTR (long-term reconstituting) activity [36]. *Jagged*-1 expression enhances HSPC self-renewal capacity [37, 38]. Recently, FGF2 was shown to expand stromal cells that may promote HSPC expansion by increasing *SCF* [13]. These results showed that FGF2 could induce niche-related gene expression and increase niche cell proliferation to support HSPCs in 5FU-damaged osteoblastic niche cells.

*FGFR* inhibition through the *FGFR* antagonist SU5402 affected HSC niche-related gene expression, including *Scf*, *Jagged-1*, and *N-cad* (Fig. 3f-h). We confirmed the expression of *Fgfrs* compared to controls in osteoblastic niche cells of mice following 5FU treatment (Fig. 4). *Fgfr1*, *Fgfr2* and *Fgfr3* were highly expressed in 5FU-treated endosteal OBs with exception of IIIc form of *Fgfr1* and *Fgfr3*. FGFR1 and FGFR2 expression in OBs have been extensively characterized. Importantly, FGFR1/2 signaling controls osteoblast gene expression and bone formation [18, 39]. Our results also indicate that *Fgfr* *III* *b* forms and *FgF2* *III* *c* might play important roles in the reception of FGF2 signals activated by 5FU-induced bone marrow suppression. We confirmed FGF signaling activation by pERK protein levels in the endosteal OBs isolated from 5FU-treated mice at the early 3-day time point (Fig. 6b). All together these results indicate that pERK activation through *Fgf* receptor upregulation increases endosteal OB proliferation after 5FU-treated OB cells received higher signal from bone marrow.

The function of OBs in proliferation and differentiation is known to relate to the HSPC supportive microenvironment [37, 40, 41]. HSC-niche factors, such as SDF-1/CXCL12 and Scf, in osteoblast lineage cells or other HSC niche cells differentially regulate HSC maintenance [19, 42]. Here, we focused on the effect of FGF2 in osteoblast, a HSC niche, recovery, and specifically the activity of endosteal osteoblastic niche cells, which showed expansion of OB layers in trabecular bone of BM following 5-FU treatment (Fig. 6c). We hypothesize that endogenous osteoprogenitor cells after BM suppression increase and then cause amplified cellularity along the endosteal surface. These amplified OBs could provide HSPC with a supportive microenvironment. It was reported that FGF2 modulates Nestin-positive mesenchymal stem cells and MS-5 murine stromal cells by generating high levels of Scf. Also, the number of BM HSPCs decreased in FGF2 KO mice. Mice treated with FGF2 affected trabecular bone growth and formation [13]. The Notch ligand Jagged-1 has been reported to increase bone formation via parathyroid hormone (PTH)-dependent PKC pathway in OBs and has important roles in stromal interactions with HSCs [37, 43]. FGF2 could maximize the anabolic effect of PTH, because FGFR signaling is also coupled to PKC pathway [18].

Since the endosteal OBs are a heterogeneous population, we examined whether the resident osteoprogenitor cells in 5FU-treated mice activated FGF signaling by increasing FGF protein levels. Conditional deletion of Osx in bone almost eliminated hematopoiesis in the BM metaphysis, and suppressing Osx expression inhibited niche generation [20, 44]. It seems that Osx-positive progenitor cells play an important role in HSC niche generation. To confirm this hypothesis, we sorted-Osx positive osteoprogenitor cells from PBS or 5FU-treated Osx-GFP mice by FACS and then performed RNA-Sequencing. HSC niche-related genes increased in the Osx + cells isolated from 5FU-treated Osx-GFP mice (Fig. 5b). *Angpt1* and *Scf* expression significantly increased following 5FU treatment. The results of RNA-Seq analysis showed that *Fgfr1–3* expression was significantly detected in 5FU-treated Osx + cells, but not *Fgfr4* (Fig. 5c-f). *Ki67* expression in Osx-positive cells from 5FU-treated mice increased at day 4 (Fig. 5g). These results indicate that the Osx + resident osteoprogenitor cells in BM can activate FGF signaling in mice under stress conditions. Similar up-regulation of Fgfr was reported in Nestin + MSCs in which the number of Nestin + MSCs that express Fgfr1 and Fgfr2 in FGF2-administered mice increased [13]. It is suggested that Osx + cells might support HSCs to allow for recovery of BM suppression via FGF signaling under stressed conditions. *Scf*, a downstream factor of FGF2, was also significantly expressed in Osx + cells following 5FU treatment.

## Conclusions

Our results indicate an important role for FGF2 in supporting HSC maintenance in stress-induced osteoblastic niche cell expansion. 5FU-treated mice induced BM suppression and showed inhibited osteoblastic niche cell proliferation with decreased *Ki67*. However, FGF2 could induce the proliferation of OBs, which express *Fgfr1–3* and highly express HSC niche-related genes during in vitro culture. FGF signaling was activated via increased pERK protein in the endosteal osteoblastic niche cells during the recovery phase. In mouse BM following 5FU, we confirmed the expansion of the OB layer in trabecular bone. HSC-niche related genes and *Fgfr1–3* were also highly expressed in Osx + osteoprogenitor cells under stress, suggesting close relation of Osx + cells in the recovery phase. In the 5FU-induced stress situation, FGF signaling could support endosteal osteoblastic niche cell proliferation as well as the reinforcement of HSC niche factors to allow for the recovery from BM HSC suppression.

## Abbreviations

5FU: 5-fluorouracil
BM: Bone marrow
EGF: Epidermal growth factor
FPKM: Fragment per kilobase of exon per million fragments mapped
H&E: Harris hematoxylin and eosin
HSPC: Hematopoietic stem and progenitor cell: FGFR: fibroblast growth factor receptor
LSK cells: Lineage negative, Sca-1 positive, cKit positive cells
LT-HSC: Long-term hematopoietic stem cells: HSC
OB: Osteoblast
